# Frugally inventive carbon fabric-based wearable sensor for monitoring human body movements

**DOI:** 10.1017/wtc.2025.10020

**Published:** 2025-07-25

**Authors:** Ahmed Alqaderi, Syed Muhammad Hafiz Syed Mohd Jaafar, Shafarina Azlinda Ahmad Kamal, Lee Hing Wah, Wei Yin Lim, Narayanan Ramakrishan

**Affiliations:** 1 Micro and Nano Devices Lab, Department of Electrical and Robotics Engineering, School of Engineering, Monash University Malaysia, Selangor, Malaysia; 2 Semiconductor R&D, MIMOS BERHAD, Kuala Lumpur, Malaysia; 3 Semiconductor and Venture Division, SIDEC SDN BHD, Shah Alam, Malaysia

**Keywords:** body movement detection, rehabilitation, strain, wearable sensors

## Abstract

We present a flexible, multilayer fabric strain sensor composed of a carbon fabric layer sandwiched between elastic bands. The sensor achieved a gauge factor of 3.4 and maintained its durability up to 635% strain. Its uniform graphite layer enabled reliable fabrication and easy integration into wearable formats. Performing well on commercial gloves and bands, the sensor effectively captured strain variations during body movement and enabled wireless transmission for real-time monitoring. Distinct resistance patterns were recorded for various body motions such as walking, jogging, jumping, and knee bending with a clear separation between high- and low-intensity activities. The overall design supports scalable fabrication and practical integration into wearable systems.

## Graphical abstract

**Figure:**
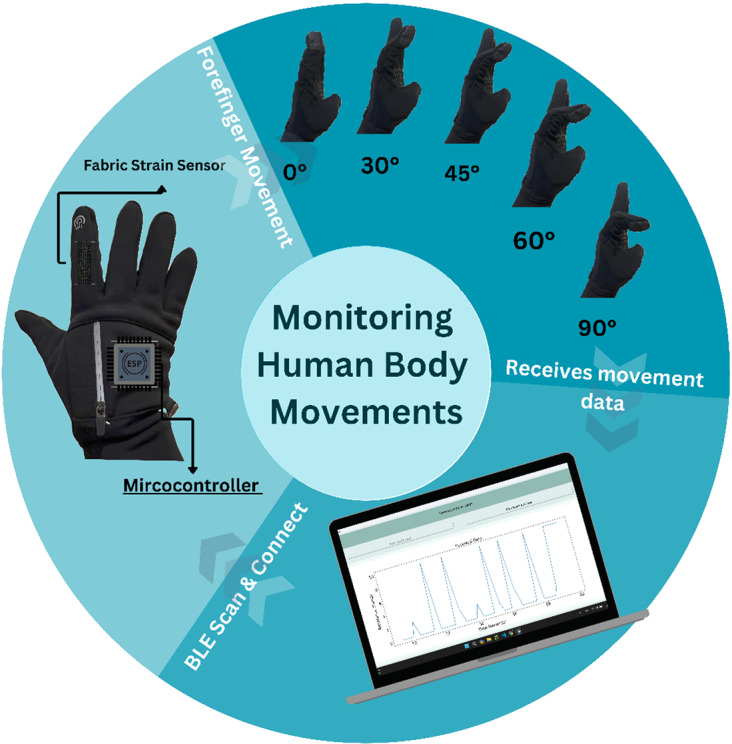

## 1. Introduction

Flexible strain sensors are widely used in wearable applications to monitor human activities, such as pulse waves, joint and wrist movements, facial expressions, and speech recognition, significantly enhancing rehabilitation, sports, and medical assistance (Shen et al., 2021; Ishii and Hirasawa, 2022; Verma et al., 2023; Zhan et al., 2023; Alkayyali et al., 2024; Zhang et al., 2024). These types of strain sensors typically consist of two primary layers: a sensing layer and a substrate layer. Among the materials used in the sensing layer, carbon is widely favored for its diverse composites, such as carbon nanotubes (CNTs) and carbon black (CB). These composites offer a variety of mechanical and electrical properties, providing enhanced sensitivity and durability, especially under harsh environmental conditions, making carbon ideal for advanced sensor fabrication (Liu et al., 2022; Jang et al., 2024; Zhang et al., 2024). On the other hand, the substrate layer is often made up of materials such as polymer nanocomposites and fabrics, selected for their ability to enhance flexibility and durability, which in turn improves the overall performance of the sensor (Hu et al., 2022; Minaminosono et al., 2023; Huang et al., 2024; Yin et al., 2024). CNT/PDMS (Liu et al., 2022; Jang et al., 2024), CB-silicone (Zhang et al., 2024), CNT powder in an acrylic elastomer (Minaminosono et al., 2023), PU/CNT composite yarn (Yin et al., 2024), wrinkled CNT film/VHB tape (Hu et al., 2022), and elastic yarn coated with LiCl (Huang et al., 2024), achieving notable sensitivity with gauge factors ranging from 1.16 to 3.3 across strain ranges of 15%–350%, demonstrating the adaptability of these materials under various conditions for wearable strain sensing applications. Although these materials offer impressive sensitivity, durability, and stretchability, scaling up their production requires multiple steps for fabrication and material preparation, such as three-roll milling, electrospinning, and electrostatic assembly – that are both costly and time-intensive (Zhang et al., 2021; Xu et al., 2022; del Bosque et al., 2023; Wang et al., 2023). This challenge highlights the critical need for a reliable flexible strain sensor that supports cost-effective mass production methods and ease for integration with wearables for efficient monitoring of human body movements. To address this need, we investigate a commercially available carbon fabric (CF)-based tape as a sensing element for developing flexible strain sensors. The fabric’s uniform graphite layers enable the fabrication of repeatable and reliable devices, while its adhesive properties allow for easy integration with flexible substrates, such as elastic bands. Copper metallic tape is used as a contact point to ensure accurate measurements. Utilizing these readily available materials not only simplifies manufacturing but also supports batch production of customized sizes and shapes suitable for wearable applications. In this work, we further examined both single- and multilayered CF sandwiched between elastic bands using stress–strain analysis, *I*–*V* characterization, and tensile testing to identify the optimal size and number of layers for achieving the highest gauge factor and strain detection range. With this optimized design, we demonstrated the CF strain sensor’s effectiveness in detecting human body movements by integrating it into a glove, knee support band, and elbow support band.

## 2. Procedure and experimental details

### 2.1. Materials required

**Figure 1.**
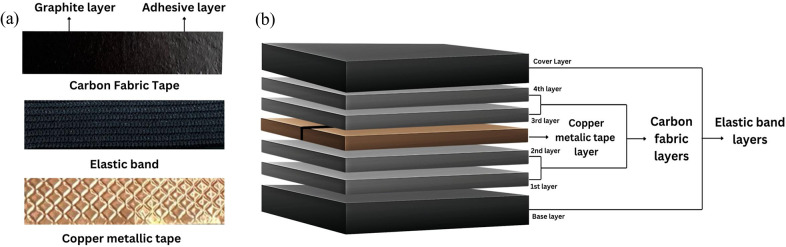
Fabrication process of a CF strain sensor. (a) The structure of the three materials used in fabricating the CF sensor, starting with CF tape, elastic band, and finally, copper metallic tape. (b) A sample of the CF strain sensor, showing its layered structure: starting with a base layer of elastic band, followed by two layers of CF, then a layer of copper metallic tape, another two layers of CF, and finally, topped with a layer of elastic band.

The CF strain sensor was fabricated using three key materials: commercial tape made of CF (Cat. No. 7313, 20 mm width 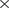 20 m, Nisshin EM Co., Ltd., Tokyo), an elastic band, and copper metallic tape, as shown in Figure 1(a). The commercial tape is made of CF,which is made of a nonwoven fabric base coated with conductive carbon powder sandwiched between acrylic adhesive layers. The elastic band made from polyester and natural latex rubber serves as the flexible substrate layer (H&T Elastic Sewing Band, DIY, Malaysia). Finally, a 10-mm copper metallic tape is used as an adhesive contact layer for measurement purposes.

### 2.2. Fabrication of carbon tape-based fabric strain sensor

The fabric strain sensor was fabricated by simple adhesive stacking and sewing methods. As shown in Figure 1(b) first four layers of CF are cut to dimensions of 5 mm 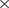 15 mm 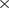 1 mm. Then, these layers are stacked together to create a two-layer CF structure of the required dimensions. Next, the elastic sewing band is cut into two pieces, each measuring 40 mm 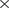 15 mm 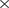 2 mm. Additionally, copper metallic tape is cut to dimensions of 10 mm 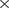 10 mm 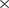 1 mm. The CF layer was then sandwiched between elastic bands with an intermediate copper metallic tape layer for electrical connections.

## 3. Results and discussion

### 3.1. Materials characterization

**Figure 2.**
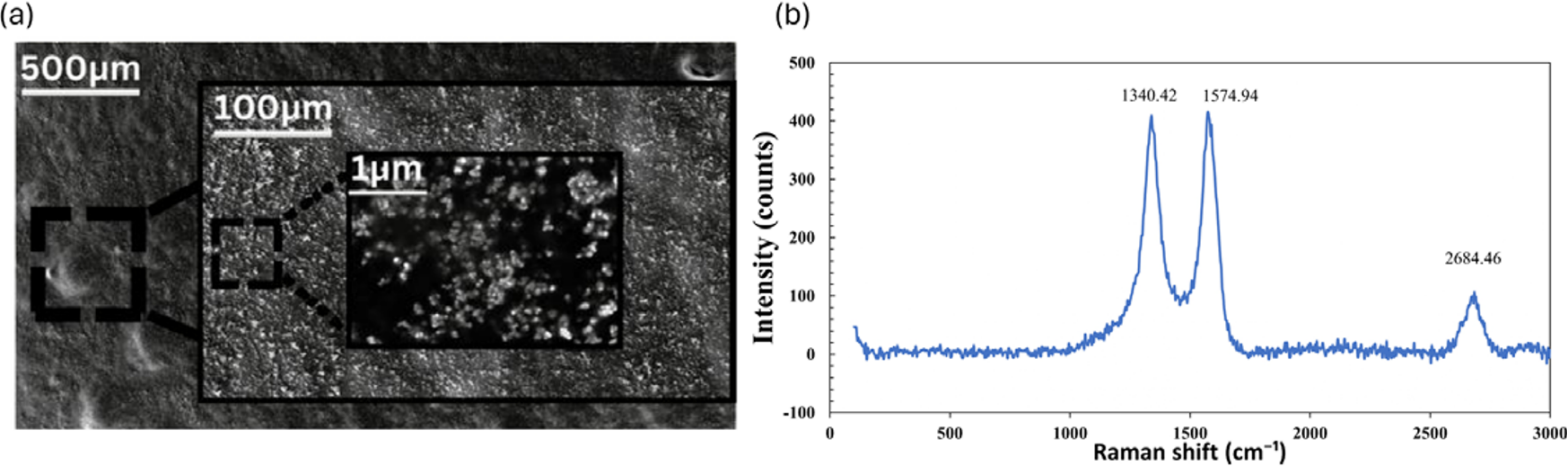
Material characterization study of the CF sensor. (a) VPFESEM images of the CF sample at magnifications of 500, 100, and 1 μm. (b) Raman spectroscopy of the CF sample.

To examine the morphological characteristics of the CF, a variable pressure field emission scanning electron microscopy (VPFESEM) analysis was conducted at three magnifications: 500, 100, and 1 μm, as shown in Figure 2(a). The VPFESEM images reveal smooth areas with conductive carbon layers and adhesive regions on the fabric. Figure 2(b) shows the Raman spectra of the sample, where a visible peak can be noticed at 1340, 1575, and 2684 cm^−1^ designated as D-band, G-band, and 2D-band, respectively, highlighting the structural properties of the graphite material. The D-band peaks indicate disorders and flaws within the carbon tape, generally associated with the breathing modes of 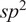 atoms in rings. The graphitic nature of the carbon tape is verified through the G-band peaks, which are due to the E2g phonon of 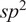 carbon atoms, representing in-plane vibrations and a crystalline structure, corroborated by FESEM imaging. The 2D band, a characteristic of the second-order Raman scattering process, provides insights into the stacking order and electronic properties of the carbon material. The position and intensity of this peak are linked to the graphene layers and the degree of stacking order in the carbon tape. These results provide a clear understanding of the structural features of carbon tape, reflecting the anticipated behavior of carbon-based materials.

### 3.2. Cost evaluation

The proposed sensor highlights a simple and cost-conscious design with pricing details as shown in Table 1; only three materials were used: CF, elastic band, and copper metallic tape, and fabrication was completed through basic steps: cutting, stacking, and stitching. Unlike other studies that rely on composite blending, chemical processing, or equipment-intensive fabrication, this method avoids surface treatment and synthesis steps entirely. All materials were used as received without any pretreatment, significantly reducing the cost and simplifying batch production. The total material cost per sensor is about USD 0.057 per sensor, further including the cost of the wireless embedded system interface and wearable fabric sums to maximum of USD 9.50, this is way lower compared to commercially available sensors. Thus, the minimal material requirement, simple integration, low production cost, and absence of complex processing steps strongly support the sensor’s frugally inventive nature.

**Table 1.** Material cost and dimension details for a single sensor unit

ID	Items	Price (USD)	Sensor dimension	Sensor cost (USD)
1	20 mm 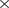 20 m, Conductive carbon double-sided tape (Nissin EM Co., Ltd.)	39.75	15 mm 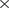 5 mm	0.039
2	10 mm 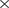 25 m, Copper foil tape with conductive adhesive (Cytron Technologies Sdn Bhd)	6.30	10 mm 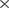 10 mm	0.005
3	(15 mm 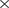 860 mm) each, H&T Elastic Sewing Band (3 pieces) (MR D.I.Y. GROUP (M) BERHAD)	0.42	15 mm 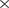 40 mm	0.013

### 3.3. Strain-sensing characterization

#### 3.3.1. Dynamic mechanical analysis

**Figure 3.**
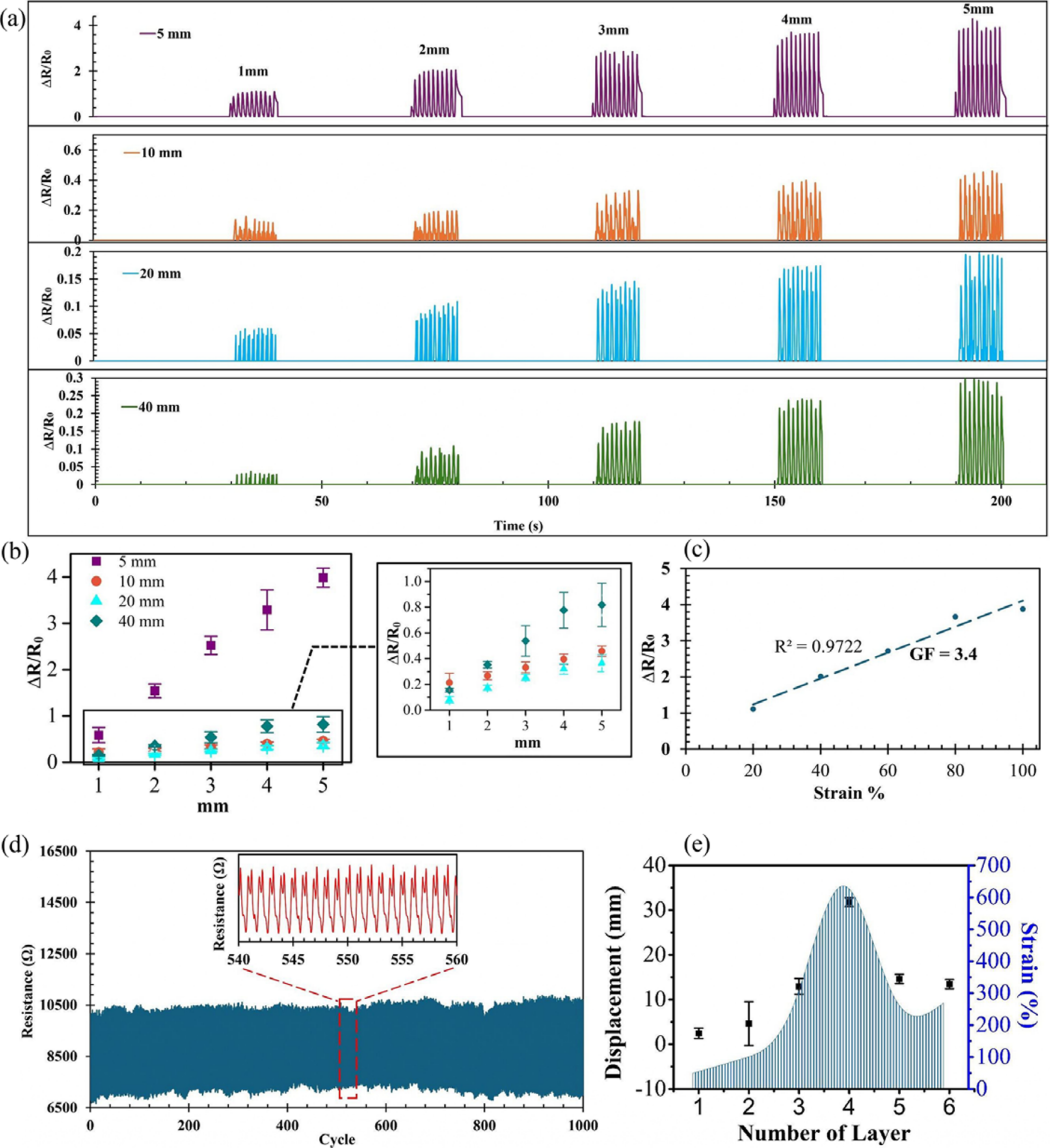
The characterization of the CF strain sensor. (a) Resistance changes for all sample sizes (5, 10, 20, 40 mm) under stretching and releasing displacements ranging from 1 to 5 mm over 10 cycles. An inset highlights the detailed response of a 2-mm stretching cycle for all samples. (b) Error bars represent the average peak resistance change over 10 cycles, based on triplicate measurements (*n* = 3). (c) Normalized resistance change 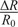 as a function of applied strain (%), demonstrating linearity and gauge factor ( 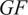 ) estimation. (d) Fatigue test results showing resistance stability over 1000 continuous stretch-release cycles at 100% strain for the 5-mm sample. (e) Displacement performance of strain sensors with different CF layer configurations, confirming the 4-layer design as the optimal configuration for balancing flexibility and tensile strength.

A series of strain tests was conducted to assess the mechanical properties and sensitivity of the proposed fabric strain sensor. First, a high-force dynamic mechanical analyzer (HDMA) (ElectroForce 3200 Series, TA Instruments/Waters Corporation, U.S.A.) was used to perform smooth cyclic stretching and releasing movements on the sensor sample, measuring the resistance changes induced by these displacements. In this setup, the strain sensor sample was secured between the instrument’s grips. Source measurement unit probe clips were attached to copper pads on the sensor sample to measure the change in resistance ( 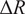 ) during each stretching and releasing cycle. 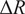 was calculated as the difference between the resistance at rest ( 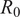 ) and the resistance under strain ( 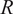 ). For this test, the HDMA instrument was set to a speed of 1 Hz (one stretch per second) and a displacement range from 1 to 5 mm. Four sample sizes – 5, 10, 20, and 40 mm – were considered for this investigation. Figure 3(a) displays the recorded time versus resistance change under different strain (displacement) levels for all measurement cycles and provides a close-up view of one cycle. Significant changes in resistance were observed for all samples, with resistance changes increasing alongside greater displacement and varying by sample size. Notably, the 5 mm sample exhibited a significantly higher 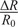 compared to other sizes, as its smaller surface area caused a more concentrated strain effect, amplifying the resistance change when stretched. This behavior results in a higher gauge factor for the 5 mm sample, as clearly demonstrated in the results shown in Figure 3(b). To evaluate these findings and identify the optimal sample size, the gauge factor ( 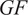 ) and linearity ( 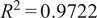 ) were calculated and assessed for repeatability and durability, as shown in Figure 3(c). To further validate the sensor’s durability, a 1000-cycle fatigue test was conducted. As shown in Figure 3(d), the sensor maintained stable resistance with minimal degradation, confirming its mechanical robustness for long-term wearable applications. 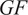 was determined using the following formula:(3.1)

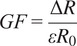

where 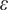 represents the strain percentage, calculated as the ratio of sample displacement to sample size, multiplied by 100. The 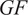 values for the 5, 10, 20, and 40 mm samples were 3.46, 0.73, 0.68, and 2.63, respectively. The 5-mm sample exhibited the highest 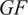 , with an average resistance ( 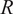 ) 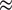 163 k 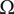 during the displacement test, due to its concentrated strain effect. 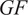 values among the different dimensions of samples can be attributed to strain distribution uniformity, filler alignment, and structural variations at different sizes, which significantly affect 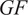 behavior (Yang and Lu, 2013; Garcia et al., 2021). Regardless, all samples withstood 100% strain due to their consistent composite structure, with the 5-mm sample demonstrating the highest stability and resilience under strain, highlighting its optimal performance for strain sensing. This part of the study further confirms that the samples produced repeatable results across a 10-cycle test at varying strain application rates.

#### 3.3.2. Tensile strength analysis

To evaluate the strength of the CF strain sensor and extract its mechanical properties, a tensile load test was conducted using the TS-2000 tensile tester (Ektron Tek Co., Ltd.). The strain test was performed at a displacement rate of 5 mm/s, with strain applied until the sensor sample either broke or could no longer endure further load. In all cases, failure occurred either due to tearing in the contact area between the copper metallic tape and the CF or a full detachment of the CF from the elastic band; the copper metallic tape itself did not tear, as it was not located in the strain-bearing region and experienced minimal deformation. This test included all sample sizes (5, 10, 20, and 40 mm) and examined different layer configurations of CF (1, 2, 3, 4, 5, and 6 layers) to determine the optimal configuration. The purpose of testing multiple layers was to investigate the trade-off between tensile strength and flexibility. The results showed that the 4-layer configuration provided a significant improvement in strength and flexibility without compromising strain response. Increasing beyond four layers added stiffness, thereby reducing flexibility. Conversely, using fewer than four layers (1–3) improved strength incrementally but did not achieve the optimal balance observed with the 4-layer configuration, as shown in Figure 3(e). Based on the tensile experiments, the 5-mm band sample with a 4-layer configuration achieved a maximum strain of 635% and a peak stress of 9.41 MPa at a displacement of 31.79 mm. This enhanced performance can be further understood by considering the role of interfacial adhesion and stress distribution within the layered structure. The stacked CF layers contributed to better mechanical cohesion and more uniform distribution of applied stress, minimizing localized strain concentrations that could otherwise lead to failure or inconsistent resistance changes. This configuration also maintained stable contact across layers during deformation, preserving the electrical path and enabling repeatable strain sensing. In contrast, fewer layers lacked the structural integrity to support higher tensile loads, while configurations beyond four layers introduced excessive stiffness, reducing stretchability and diminishing the sensor’s responsiveness to strain. Therefore, the 4-layer design represents a balanced structure that effectively combines flexibility, strength, and electrical reliability for wearable-sensing applications. A summary of the mechanical parameters of the 4-layer configuration is provided in Table 2. These results confirm that CF strain sensors with a 4-layer configuration can withstand high loads, strain, and stress while remaining flexible and ductile. The performance of the proposed sensor was also bench marked against recently reported carbon-based strain sensors and is presented in Table 3. The superior strain range and sensitivity presented can be attributed to two key design features: the use of a highly stretchable elastic band and a multilayer CF structure. This combination enables the sensor to endure extensive mechanical deformation while maintaining stable electrical performance. Additionally, the uniform graphite coating on the CF ensures repeatable and consistent resistance changes across large strain values, contributing to a stable gauge factor. Unlike more complex fabrication methods involving chemical modification or composite blending, the simplicity of our structure reduces variability and enhances scalability. These factors collectively explain the higher strain range and sensitivity achieved in our work compared to previous studies.

**Table 2.** Tensile strain sensing results for CF strain sensor

Parameters	Values
Peak load	141.1 N
Peak stress	9.4 MPa
Stiffness	4.439 N/mm
Yield point extension	31.79 mm
Strain at maximum load	635%
Elongation at break	127%
Young’s modulus	1.479 MPa
Gauge factor ( 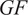 )	3.4

**Table 3.** Comparison of carbon-based strain sensors reported in the literature

ID	No. of materials	Materials	No. of fabrication Steps	Resistance range	Strain range	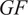	Linearity	Cycles	References
1	3	Carbon nanotubes (CNTs), silica aerogel, and PDMS	6	35%	30.00%	1.16	0.99	–	Jang et al. (2024)
2	–	–	–	–	100.00%	1.34	0.99	1000	Zhang et al. (2024)
3	4	Acrylic elastomer, MWCNTs, Suave–10F100, and VHB elastomers	5	200%	125.00%	1.46	0.99	50	Minaminosono et al. (2023)
4	7	PU, CNTs, DMF, THF, sulfuric acid, nitric acid, and glycerol	4	150%	350.00%	1.7	Nonlinear	1000	Yin et al. (2024)
5	4	CNTs, Tegaderm film, VHB elastomer tape, and silver paste	4	–	300.00%	2.07	0.99	1000	Hu et al. (2022)
6	6	CNT, elastic yarn, LiCl, filter paper, latex tube, and copper metallic tape	7	–	100.00%	2.84	Nonlinear	1000	Huang et al. (2024)
7	7	PDMS, CNT, silicone oil, perfluorinated silane, xylene, Krytox, paraffin oil	7	270%	100.00%	3.3	Nonlinear	500	Liu et al. (2022)
8	3	Carbon fabric, copper metallic tape, elastic band	3	276%	635.00%	3.4	0.97	–	This work

### 3.4. Temperature and humidity

**Figure 4.**
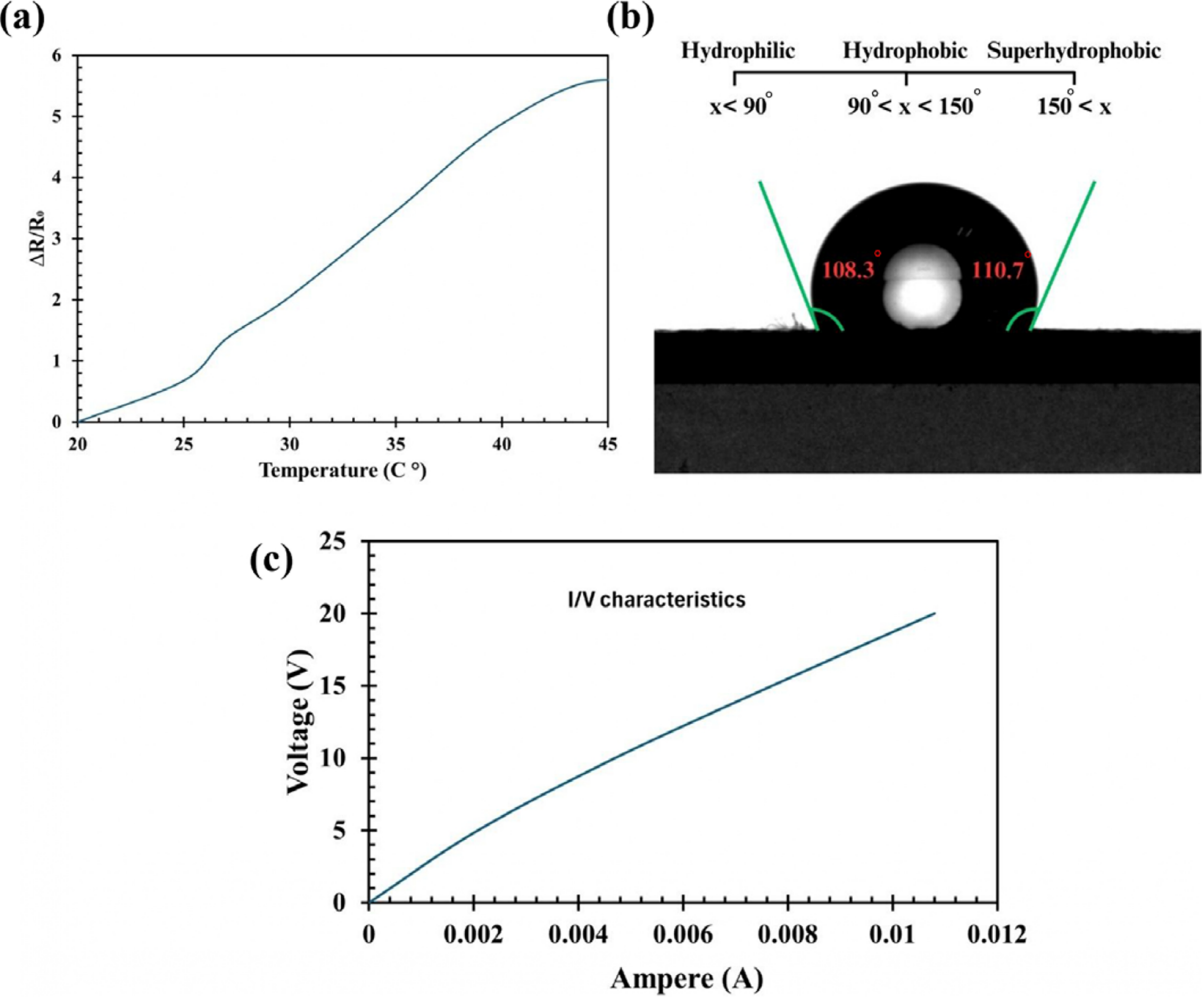
Temperature and I–V characteristics of the CF strain sensor. (a) Observational study of resistance changes in the CF strain sensor from 20°C to 45°C, with a maximum change of 5.75% at 45°C. (b) Hydrophobicity test results of the 4-layer CF structure, showing contact angles of 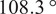 (left) and 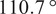 (right), confirming hydrophobic behavior. (c) Observational study of I-V characteristics of the CF strain sensor, showing a resistive and linear response from 0 to 20 V.

To further investigate the effects of temperature and humidity, the CF strain sensor was mounted on a microcomputer-controlled hot plate (BY1010, Bangye Electronics, China), and resistance changes were measured as the temperature varied from 20 °C to 45 °C. This temperature range was chosen to reflect typical room temperature conditions. Figure 4(a) shows the change in resistance relative to applied temperature, with a maximum resistance change of approximately 5.75% resistance change observed at 40 °C. Despite these effects, the sensor exhibited only a minor sensitivity to temperature within this range, and the change in resistance was negligible compared to that observed under applied strain. This slight increase in resistance can be attributed to the positive temperature coefficient (PTC) behavior of carbon black-based composites, where the conductive network between particles gets disrupted as temperature rises, leading to increased resistivity (Xu et al., 2022). Additionally, the humidity resilience of the sensor is supported by the hydrophobic test carried out on the 4-layer carbon tape structure used in the sensor, which showed hydrophobic behavior with a contact angle of 108.3*°* ( 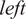 ) and 110.7*°* ( 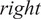 ) both exceeding the 90*°* threshold for hydrophobic surfaces as shown in Figure 4(b). These values indicate that moisture is unlikely to penetrate or affect the conductive layers, reinforcing the sensor’s stable performance under high humidity conditions (Wei et al., 2023).

### 3.5. I–V characteristics

Finally, the 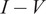 characteristics of the CF strain sensor were studied using a source meter (2450 SourceMeter®, Keithley). Figure 4(c) shows the voltage versus current, revealing that the sensor is resistive and exhibits a linear response within the voltage range of 0 to 20 *V.* This linearity indicates the ease of further instrumentation for constructing wearable devices to monitor human body movements.

## 4. Human body movement monitoring applications

**Figure 5.**
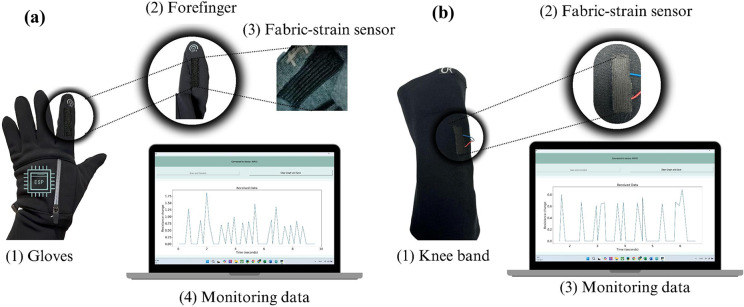
Implementation of CF strain sensors in human monitoring applications: (a) commercial gloves embedded with fabric strain sensors on the forefinger. (b) Knee band embedded with fabric strain sensors for movement detection.

**Figure 6.**
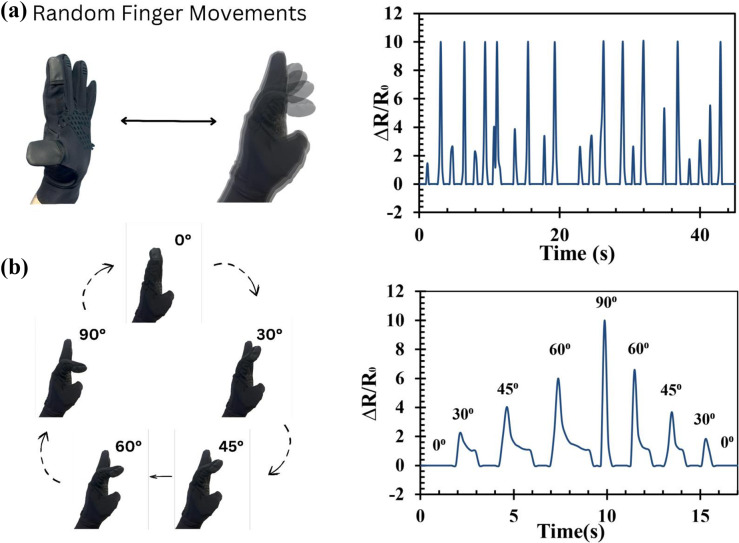
Commercial glove with forefinger movement monitoring: (a) monitoring the movement of random forefinger bending. (b) Monitoring the angle movements of the forefinger.

To implement the fabric strain sensor as a device for detecting human body movements, the sensor was embedded in two types of wearables: (1) a hand glove and (2) knee and elbow bands, as shown in Figure 5(a) and (b), respectively. To enhance the function, sensors were interfaced with an ESP32 module (Seeed Studio Xiao C3), and the change in resistance values caused by body movements was transmitted wirelessly to a terminal computer via Bluetooth communication. The sensors and the interface chip were stitched with wearable fabric to complete the wearable sensor. Further experiments were conducted to measure the change in resistance for finger movements: (1) flexion, involving movement of the finger toward the palm, and (2) extension, moving it away from the palm. Figure 6(a) shows a typical wireless operation of the sensor, with a recorded video available in the Supplementary Materials, and Figure 6(b) shows the change in angles of finger movements.

As seen, the recorded analog signals from the sensor at the computer terminal during typical finger movements demonstrated good repeatability and responsiveness in detecting finger movements.

**Figure 7.**
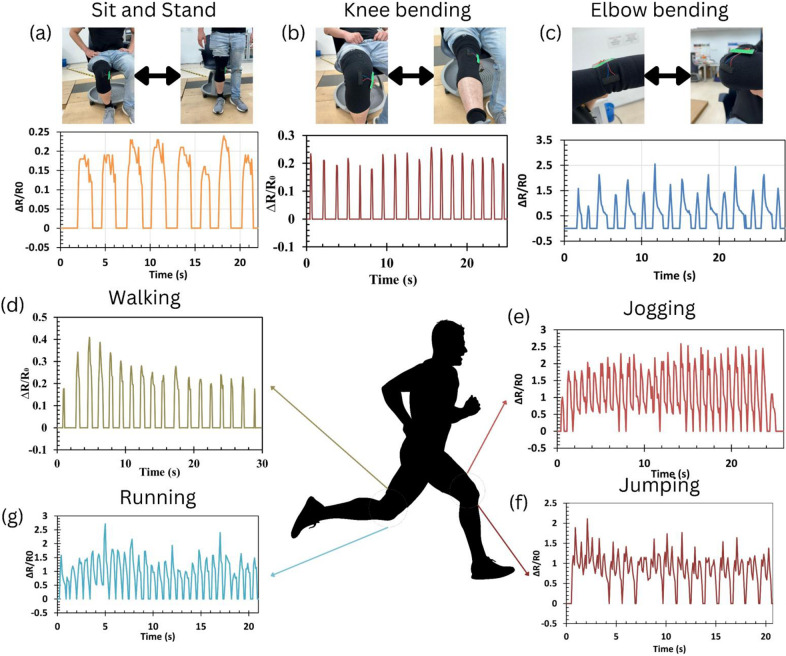
Implementation of the CF strain sensor in human monitoring applications across different body parts and sports activities. For example, sit-and-stand movements and knee bending (a–b) captured a maximum resistance change of 0.25. Additionally, various sport actions, such as elbow bending (c), walking (d), jogging (e), jumping (f), and running (g), were monitored and recorded maximum resistance changes of 2.5, 0.4, 2.5, 2.25, and 2.75, respectively.

Further, for the band configuration, the wearable was worn on the elbow and knee and monitor strain changes were measured for the body movements. Figure 7 shows the recorded sensor signals for different body parts in different movement activities, such as (a) sit-and-stand movements, (b) knee bending, (c) elbow bending, (d) walking, (e) jogging, (f) jumping, and (g) running, which can be seen that the recorded ratio of change in resistance was in the range of 0.25, 0.25, 2.5, 0.4, 2.5, 2.25, and 2.75, respectively.

**Figure 8.**
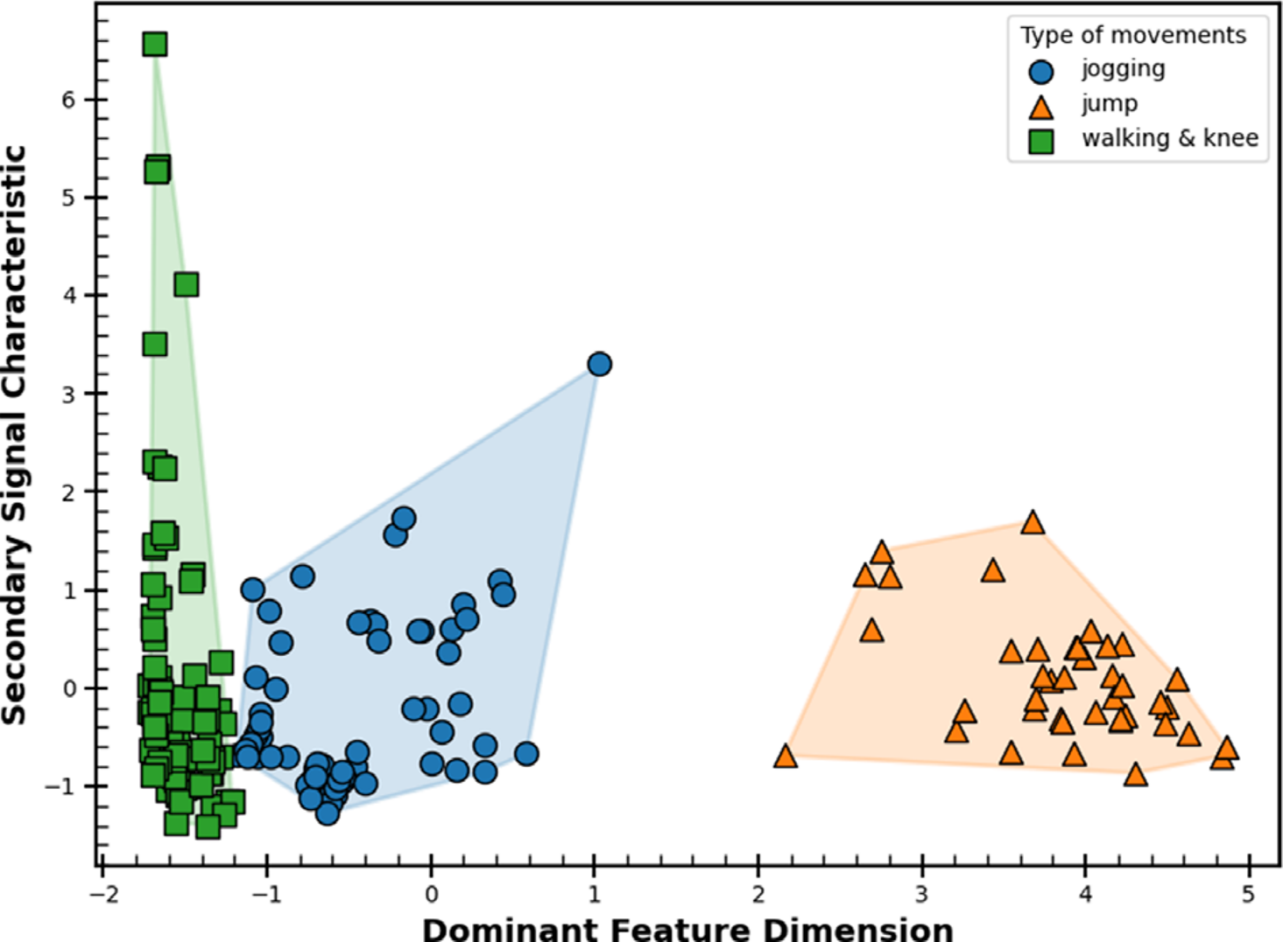
PCA cluster plot indicating movement types using extracted resistance features.

It can be realized from these results that different body movements show distinct values patterns allowing one to monitor movements digitally. Overall, this demonstration indicates the feasibility of measuring important body movements that will be useful during medical rehabilitation monitoring and other sports training activities. More importantly, the fabric carbon strain sensor is a frugal solution for batch fabrication and easy integration with commercial wearable. To further visualize clusters within the sensor response dataset, principal component analysis (PCA) was conducted and cluster plots generated using Python 3.12 software. Figure 8 presents a PCA-based cluster plot showing a clear division between leg-related movements: jogging, jumping, and a combined group of knee bending and walking. The knee bending and walking movements were merged due to their strong similarity in motion pattern and frequency characteristics. In addition, standing and running were excluded from the analysis to avoid overlap with other movements, particularly jogging and knee bending. The separation between these movement groups is driven by extracted signal features such as mean value, standard deviation, peak-to-peak amplitude, skewness, and kurtosis, all of which proved highly suitable for pattern recognition. This outcome highlights the potential for future studies to implement machine learning classification models with larger datasets, aiming to enhance prediction accuracy and enable adaptive feedback systems.

## 5. Conclusion

We proposed a frugally inventive fabric-carbon strain sensor with simple fabrication steps, yet robust for precise strain measurement. The precise design of the CF integrated with an elastic band allows maximum stretchability and durability, making it suitable for wearable applications. The sensor was rigorously tested on a dynamic mechanical analyzer, performing cyclic stretching and releasing movements across different-sized samples, which yielded a gauge factor of 3.4 within the 100% strain range. Additionally, tensile testing showed that the proposed band sensor could endure up to 141.1 N and achieve up to 635% strain at maximum load, demonstrating impressive durability and repeatability – key attributes for wearable device applications. The performance of the proposed sensor was benchmarked with recently reported carbon-based strain sensors, and the strain and resistance change ranges were significantly above the reported values while requiring the minimal number of materials and fabrication steps. This clearly indicates the novelty and frugal innovation of the proposed sensor. Finally, the sensor was interfaced with an ESP32-based wireless system and tested in different wearable configurations such as hand glove, elbow, and knee support bands. The demonstrations clearly confirmed the sensor’s capability to measure body movements, showing distinct resistance changes enabling easy identification and measurement. In conclusion, these tests and the cost-effective design confirm that the CF strain sensor can be mass-produced for diverse wearable applications, effectively measuring and monitoring human motion in medical and sports fields.

## Data Availability

The data that support the findings of this study are available from the corresponding author, N. Ramakrishnan, upon reasonable request.
